# Supplementary material for: The Resonance and Adaptation of Neurospora crassa Circadian and Conidiation Rhyth ms to Short Light-Dark Cycles
Source: J Fungi (Basel). 2021 Dec 29;8(1):27. doi: 10.3390/jof8010027 (PMC8780863; doi:10.3390/jof8010027)
Supplement: Supplementary file 1 [file jof-08-00027-s001.zip › File S1.pdf]

File S1

**Supporting Information**<br><br>Contents	Description
Supplemental Table 1	*Neurospora crassa* strains used in this study
Supplemental Table 2	Parameters of the mathematical model
Supplemental Table 3	The growth rates and periods of conidiation rhythms of indicated *Neurospora crassa* strains
Supplemental Figures	Fig. S1-6
Supplemental methods	Protocol and parameters for modeling
	RNA-seq methods and parameters
Supplemental references	15 cited articles

Table S1. *Neurospora crassa* strains used in this study

Strain	Genotype	Source/reference
WT	*mata*, *ras-1*^{bd}	Laboratory stock
Wild-type	*matA, 74A-OR23-1A (#4200)*	FGSC4200
wc-1^{RIP}	*ras-1*^{bd}, *wc-1*^{RIP}	Laboratory stock
Δ*wc-2*	*matA*, *ras-1*^{bd}, Δ*wc-2*^{bar}	Laboratory stock
cog-1	*mata*, *ras-1*^{bd}, *cog-1*	(Nsa *et al.* 2015)
Δ*cry*	*mata*, *ras-1*^{bd}, Δ*cry*^{hyg}	Laboratory stock
Δ*vvd*	*mata*, Δ*vvd*^{hyg}	FGSC11555
Δ*nop-1*	*matA* ,Δ*nop-1*^{hyg}	FGSC15897
wcc^{DKO}	*ras-1*^{bd}, *wcc*^{dko}	Laboratory stock
frq^{10}	*ras-1*^{bd}, *frq*^{10}	Laboratory stock
fwd-1^{RIP}	*ras-1*^{bd}, *fwd-1*$^{RIP,his-1}$	Laboratory stock
Δ*phy-1*	*matA*, *phy-1*^{AF1}	(Chen *et al.* 2009)
Δ*phy-2*	*mata*, *phy-1*^{AF1}*, phy-2* ^{AF1}	(Chen *et al.* 2009)
Δ*phy-1*, Δ*phy-2*	*mata*, *phy-1*^{AF1}*, phy-2* ^{AF1}	(Chen *et al.* 2009)
Δ*cry*, *cog-1*	*matA*, Δ*cry*^{hyg}, *cog-1*	(Nsa *et al.* 2015)
Δ*vvd*, *cog-1*	*matA*, *ras-1*^{bd}, Δ*vvd*^{hyg}, *cog-1*	(Nsa *et al.* 2015)
Δ*wc-1*, *cog-1*	*matA*, *ras-1*^{bd}, Δ*wc-1*^{bar}, *cog-1*	(Nsa *et al.* 2015)
Δ*cry*, *cog-1*	*matA*, *ras-1*^{bd}, Δ*cry*^{hyg}, Δ*vvd*^{hyg}	(Nsa *et al.* 2015)
Δ*cry*, Δ*wc-1*	*matA*, *ras-1*^{bd}, Δ*cry*^{hyg}, Δ*wc-1*^{bar}	(Nsa *et al.* 2015)
Δ*vvd*, Δ*wc-1*, *cog-1*	*matA*, *ras-1*^{bd}, Δ*vvd*^{hyg}, Δ*wc-1*^{bar}, *cog-1*	(Nsa *et al.* 2015)
Δ*cry*, Δ*vvd*, *cog-1*	*matA*, *ras-1*^{bd}, Δ*cry*^{hyg}, Δ*vvd*^{bar}, *cog-1*	(Nsa *et al.* 2015)
Δ*cry*, Δ*vvd*, Δ*wc-1*	*matA*, *ras-1*^{bd}, Δ*cry*^{hyg}, Δ*vvd*^{hyg}, Δ*wc-1*^{bar}	(Nsa *et al.* 2015)
Δ*cry*, Δ*wc-1*, *cog-1*	*matA*, *ras-1*^{bd}, Δ*cry*^{hyg}, Δ*wc-1*^{bar}, *cog-1*	(Nsa *et al.* 2015)
Δ*cry*, Δ*wc-1*, Δ*vvd*, *cog-1*	*matA*, *ras-1*^{bd}, Δ*cry*^{hyg}, Δ*wc-1*^{bar}, Δ*vvd*^{hyg}, *cog-*	(Nsa *et al.* 2015)
frq2	*ras-1*^{bd}, *frq*^{2}	(Feldman and Hoyle. 1973)
frq7	*ras-1*^{bd}, *frq*^{7}	(Gardner and Feldman 1980)

Table S2 Parameters of the mathematical model

Parameters	Value	Parameters	Value
$\mathbf{k_{smfrq}}$	1	K1	1.8
$\mathbf{k_{smfrq2}}$	6	K2	0.5
$\mathbf{k_{sFRQ_n}}$	0.4	K3	5
$\mathbf{ks_{basal}}$	0.2	K4	0.02
$\mathbf{ks_{wcc}}$	0.2	K5	1
**k_wcc2la**	0.001	K6	0.18
**k_la2wcc**	0.001	kd1	1
**kd_lawcc**	5	kd2	0.6
$\mathbf{k_{svvd}}$	1	kd3	0.18
**k_mwcc**	0.5	kd4	0.02
**n1**	2	kd5	0.05
**n2**	2	L	0, when dark
**τ**	5		0.2, when light
**k_frq2wcc**	1		

Table S3. The growth rates and periods of conidiation rhythms of indicated *Neurospora crassa* strains

**Strain**	**Conidiation period in DD**	**Growth length in 24 h (cm)**		
frq2	18.3±0.1h	*T* cycles	*T* = 18 h	4.2±0.2
			T = 24 h	4.1±0.1
			T = 27 h	4.1±0.1
frq7	26.5±0.2h	*T* cycles	*T* = 18 h	3.8±0.2
			T = 24 h	3.6±0.1
			T = 27 h	4.1±0.1
301-5 (WT)	22.1±0.3h	*T* cycles	*T* = 18 h	3.9±0.2
			T = 24 h	3.8±0.2
			T = 27 h	4.2±0.2

Data are mean ± SD (n=3). Table S3 is related to Fig. 1B.

Fig. S1

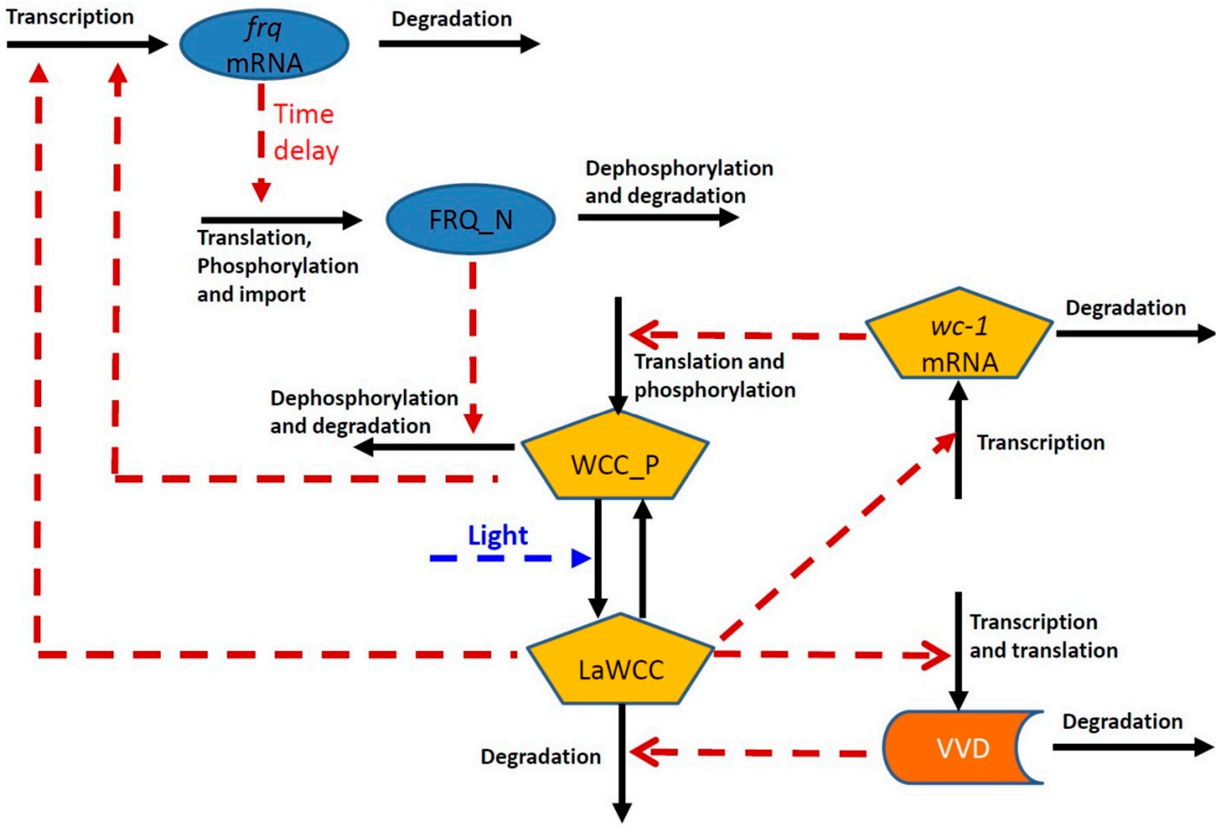

Figure S1 | Scheme for the mathematical model of *Neurospora* circadian rhythm. The core negative feedback loop drives the autonomous oscillation of circadian clock, in which *frq* is negatively auto-regulated by inducing the degradation and phosphorylation of WCC. In addition, light depended WCC (LaWcc) promotes the transcription of VVD. In return, VVD accelerates the degradation of light depended WCC, which closes another negative feedback loop. Light can affect the circadian clock by inducing the transfer rate from phosphorylated WCC to light depended WCC.

Fig. S2

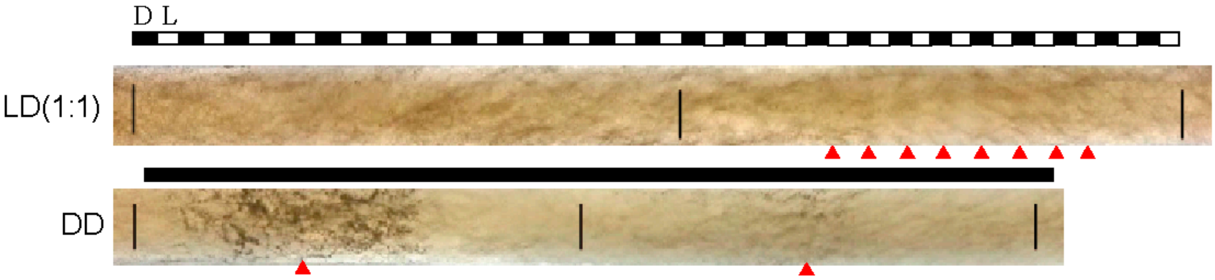

FIGURE S2 | Conidiation rhythms of the FGSC4200 strain in LD1:1 and DD conditions. Representative results (n ≥ 3) are shown. The strains were grown under white light and the light intensity was 5000 lux. The colors were inverted for better observation of the conidiation details.

Fig. S3

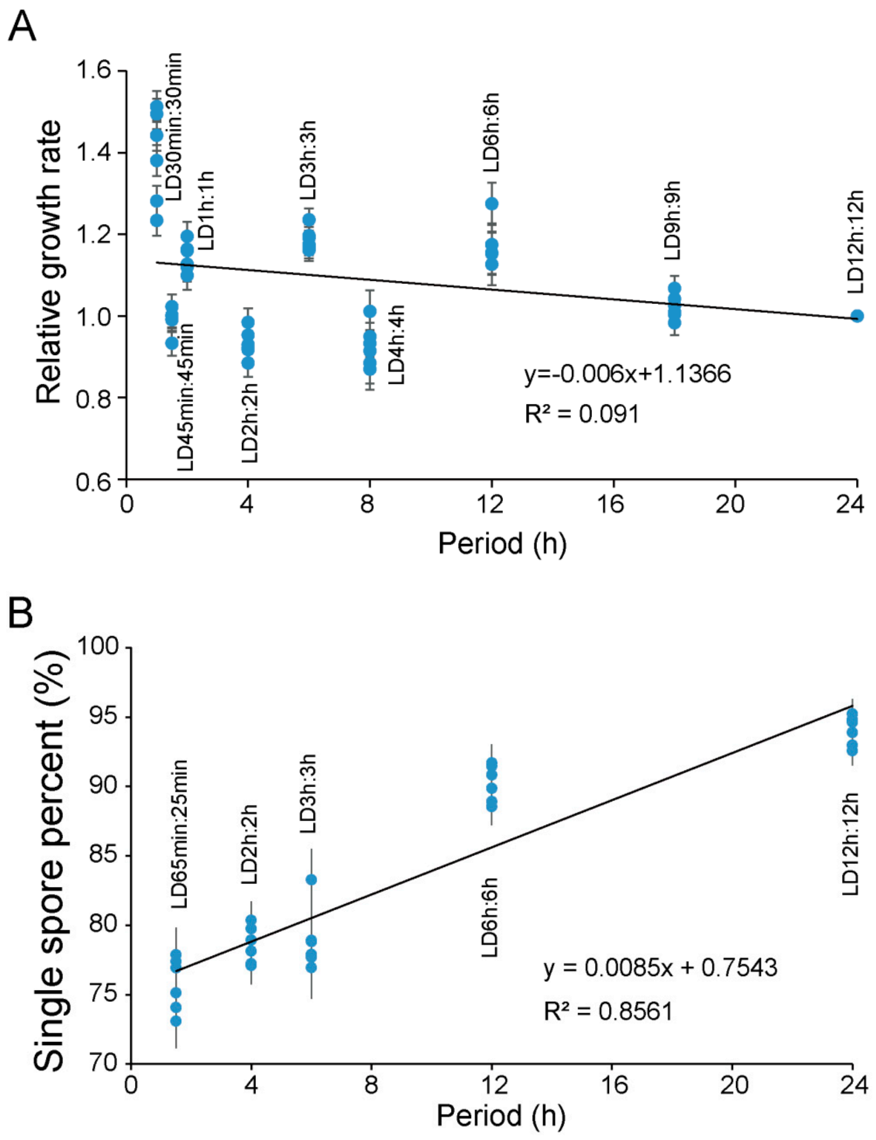

FIGURE S3 | Coefficient analysis between the LD cycles and growth rates (A) and between the LD cycles and microconidia proportion (B). The daily growth lengths were normalized to the data in LD12:12 (A) and the proportions of microconidia were the percentages of the microconidia quantity divided by the total quantity of both microconidia and macroconidia (B). Bivariate poisson regression model was used to calculate the coefficient. The results suggest that the LD cycle is correlated with microconidia proportion but not the growth rate.

Fig. S4

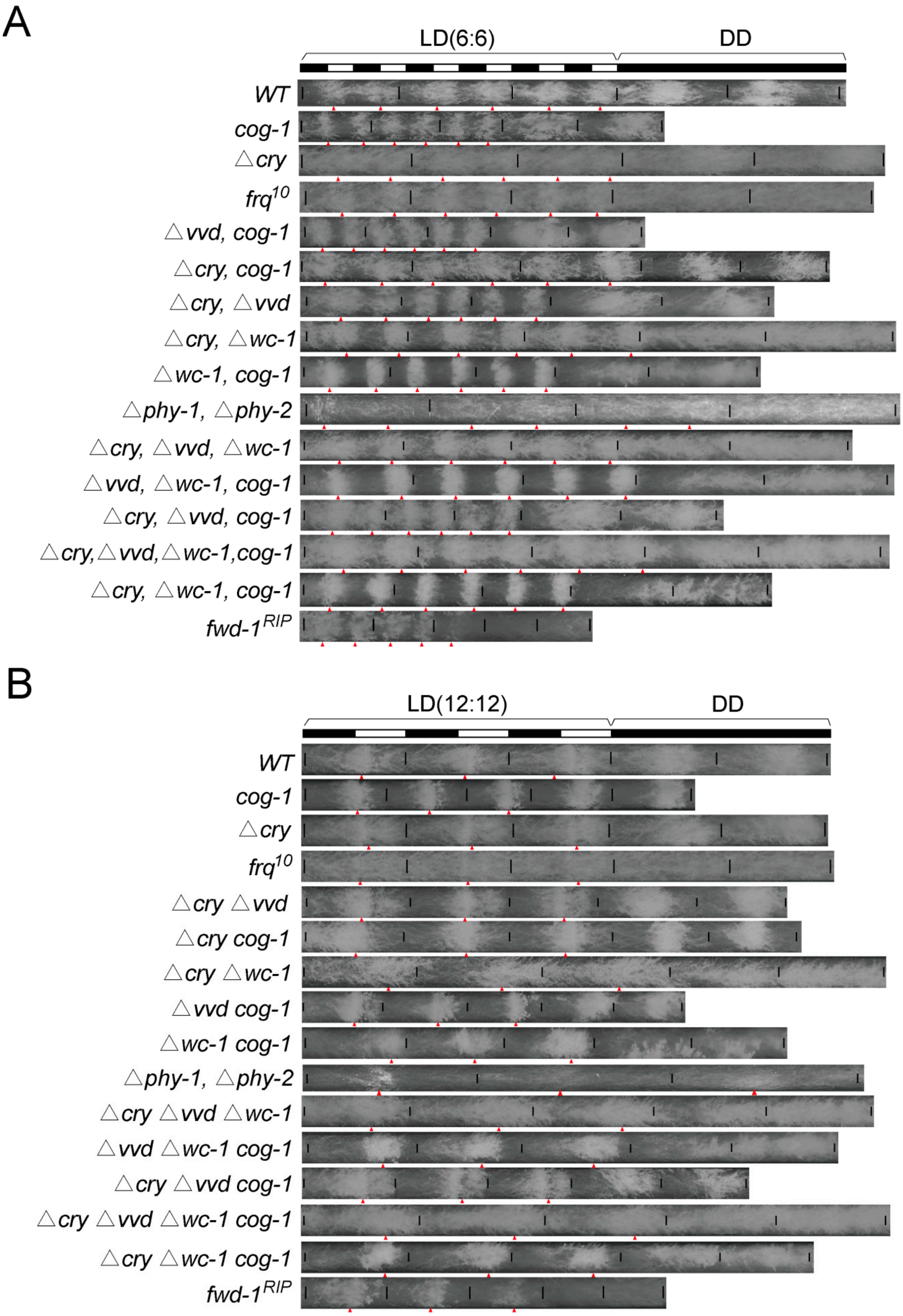

Figure S4 | Impacts of light sensors on the adaptation to short LD cycles. Race tube results of indicated strains under indicated LD cycles including LD6:6 **(A)** and LD12:12 **(B)**. Representative results (n ≥ 3) are shown. The strains were grown under white light and the light intensity was 5000 lux.

Fig.S5

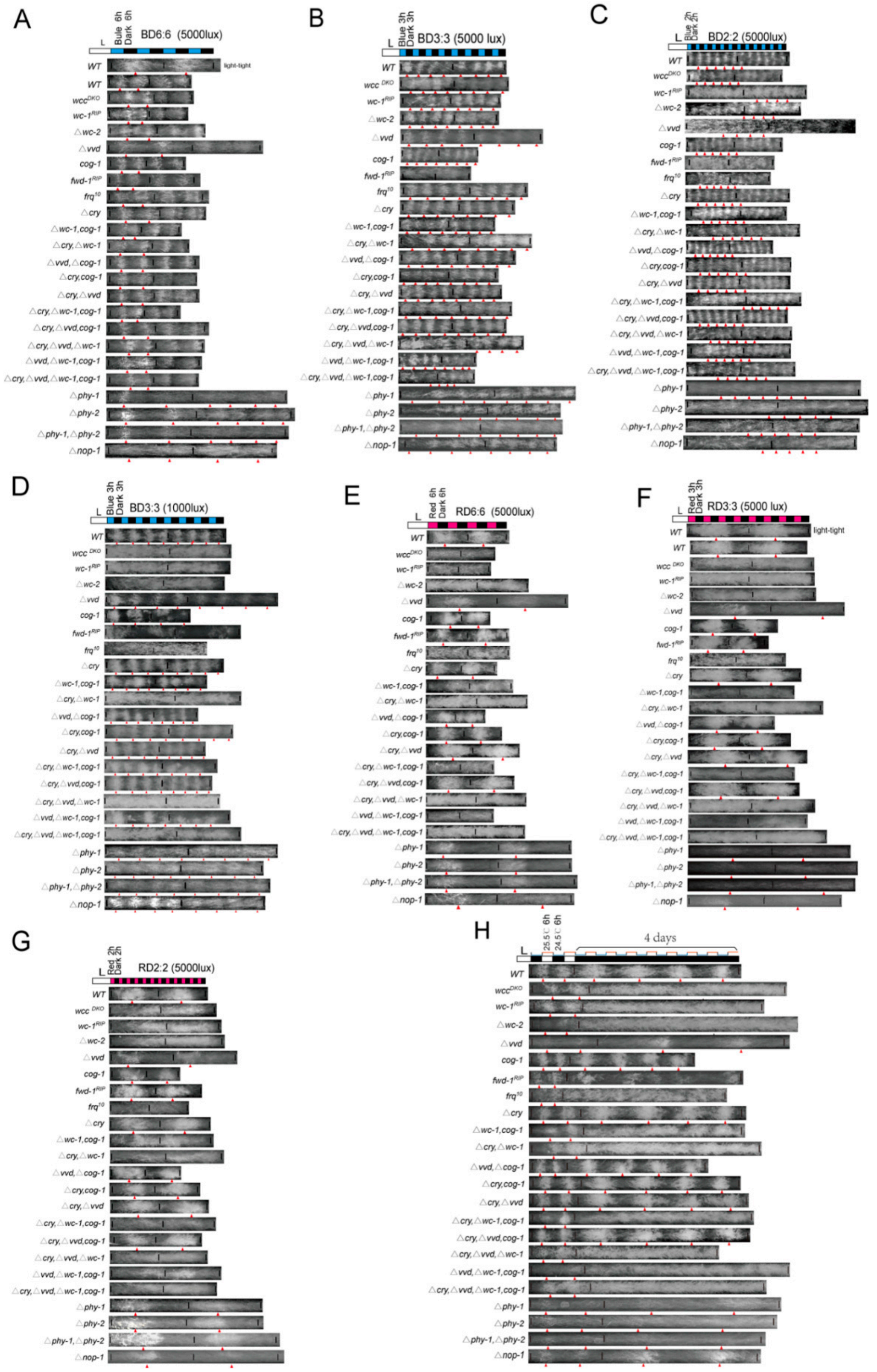

Figure S5 | Conidiation rhythms of *Neurospora* strains in short blue light/dark (BD) and red light dark (RD) cycles. **(A-G)** The conditions were BD6:6 **(A)**, BD3:3 **(B)**, BD2:2 **(C)**, BD3:3 **(D)**, RD6:6 **(E)** and RD2:2 **(G)**, respectively. The light intensity was 5000 lux or 1000 lux as indicated. **(H)** The race tube results of indicated strains grown under temperature cycles (25.5℃6h: 24.5℃6h) for 4 days. Representative results (n ≥ 3) are shown. Triangles in red denote the conidiation bands.

Fig. S6

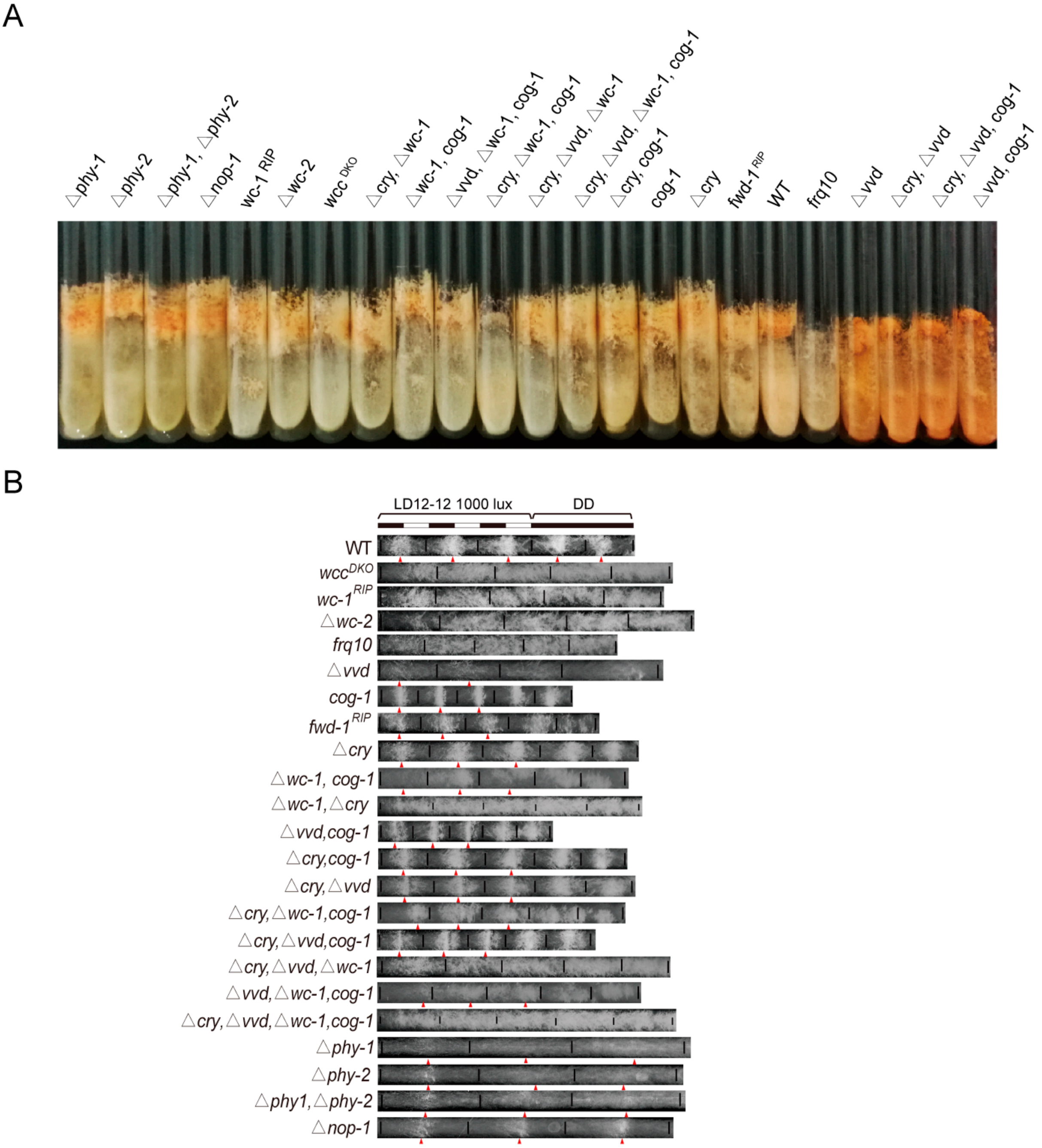

FIGURE S6 | Growth phenotype and conidiation rhythms of indicated strains. **(A)** Different mycelia color of indicated strains grown on slants showing. Representative results (n≥3) are shown. The strains were grown under white light and the light intensity was 5000 lux. **(B)** Conidiation rhythms in low white light (1000 lux). A,B: Race tube results of conidiation rhythms of indicated strains in LD12:12. Triangles denote the conidiation bands.

Supplemental protocol and parameters for modeling

In the past few years, a number of mathematical models of *Neurospora* circadian rhythm have been built (Goldbeter, 1995; Glossop et al., 1999; Gonze and Goldbeter, 2000; Gonze et al., 2000; Gin et al., 2013). Gonze et al constructed a 3-varible mathematical model to describe the dynamics of *frq* and explained the mechanism of autonomous oscillation in *Neurospora* circadian clock (Gonze et al., 2000). Furthermore, Elan et al. presented a detailed model to explore the entrainment of *Neurospora* circadian clock by the light (Gin *et al.* 2013). Here, we proposed a simpler model to explore how the *Neurospora* circadian clock responses to different light dark cycles.

The mathematical model schematized in Fig S1 can be turned into differential equations. As shown in Fig S1, the transcription of *frq* can be positively regulated by the phosphorylated WCC (denoted wccp) and light dependent WCC (denoted lawcc). Therefore the concentration of *frq* mRNA (mfrq) can be determined by the following equation.

$$\frac{\mathrm{dmfrq}}{\mathrm{dt}} = \mathrm{k_{smfrq}} * \frac{\mathrm{wccp^{n1}}}{\mathrm{wccp^{n1}} + \mathrm{K1^{n1}}} + \mathrm{k_{smfrq2}} * \frac{\mathrm{lawccp^{n1}}}{\mathrm{lawccp^{n1}} + \mathrm{K6^{n1}}} - \mathrm{kd1} * \mathrm{mfrq}$$

After that, *frq* mRNA is translated into the corresponding protein and FRQ protein further enter the nucleus (denoted FRQn). Since some intermediate biological processes are required for translation and the entry of the FRQ protein into the nucleus, the concentration of the nucleic FRQ protein is determined τ hours ago.

$$\frac{\mathrm{dFRQ_n}}{\mathrm{dt}} = \mathrm{k_{sFRQ_n}} * \mathrm{mfrq}(\mathrm{t} - \tau) - \mathrm{kd2} * \mathrm{FRQ_n}$$

The regulation of *wc-1* mRNA (mwcc) is also involved in this model.The transcription of *wc-1* can be promoted by the light dependent WCC. Thus, we can use the following equation to describe the variation of *wc-1* mRNA.

$$\frac{\mathrm{dmwcc}}{\mathrm{dt}} = \mathrm{k\_mwcc} * \frac{\mathrm{lawcc^{n1}}}{\mathrm{lawcc^{n1}} + \mathrm{K5^{n1}}} - \mathrm{kd4} * \mathrm{mwcc}$$

Following the translation of *wc-1* mRNA into the corresponding protein, we considered two states of WCC complex: phosphorylated and light dependent. The degradation of the phosphorylated WCC can be induced by the nucleic FRQ protein. Therefore, a core negative feedback loop is formed between *frq* and phosphorylated WCC (Schafmeier et al., 2005; He et al., 2006). Also, based on the experimental data, VVD can promote the degradation of light dependent WCC. The two states of WCC complex can transfer to each other.Inaddition, light can accelerate the transfer rate from the phosphorylated WCC complex to light dependent form.

$$\frac{dwccp}{dt} = ks_{basal} + ks_{wcc} * mwcc - k_{frq2wcc} * \frac{{FRQ_n}^{n2}}{{FRQ_n}^{n2} + K2^{n2}} * wccp - (L + k_{wcc2la}) * wccp + k\_la2wcc * lawcc$$

$$\frac{dlawcc}{dt} = L * wccp + k\_wcc2la * wccp - k\_la2wcc * lawcc - kd\_lawcc * \frac{vvd^{n1}}{vvd^{n1} + K3^{n1}} * lawcc - kd5 * lawcc$$

We also introduce VVD into the model, which forms a negative feedback loop with light depended WCC. VVD promote the degradation of light depended WCC as we mentioned above. In return, the light depended WCC positively regulate VVD (Hunt et al., 2010; Chen et al., 2010; Malzahn et al., 2010). Thus, the concentration of VVD can be determined by the following equation.

$$\frac{dvvd}{dt} = k_{svvd} * \frac{lawcc^{n1}}{lawcc^{n1} + K4^{n1}} - kd3 * vvd$$

RNA-seq methods and parameters

The isolated RNA samples isolated from duplicates (repeat #1, repeat #2) were processed to prepare the mRNA-seq library using the standard Illumina protocol. The sequencing was performed on an Illumina HiSeq 2000 at Forevergen, Guangzhou, China. The number of clean reads (repeat #1, repeat #2) of strains (△*cry*,△*vvd*,△*wc-1*) at DD24h, (△*cry*,△*vvd*,△*wc-1*) 45 min after light pulse (1000lux), (△*cry*,△*vvd*,△*wc-1*) 45 min after light pulse (5000 lux). The mapping ratios of these strains (replicates) were: (79.2%, 78.5%), (80.3%, 80.4%), (81.5%,79.6%), respectively. Tophat was used as the aligner to map the reads to the reference genome [*N. crassa* OR74A (NC12)]. Cufflinks was used to reconstruct the transcripts and estimate the gene expression levels (Trapnell et al., 2010).

REFERENCES

Chen, C.-H., DeMay, B.S., Gladfelter, A.S., Dunlap, J.C., Loros J.J., 2010. Physical interaction between VIVID and white collar complex regulates photoadaptation in Neurospora. Proc. Natl. Acad. Sci. U S A 107, 16715-16720.

Chen, C.-H., Ringelberg, C.S., Gross, R.H., Dunlap, J.C., Loros, J.J., 2009. Genome-wide analysis of light-inducible responses reveals hierarchical light signalling in Neurospora. EMBO J. 28, 1029-1042.

Feldman, J.F., Hoyle, M.N., 1973. Isolation of circadian clock mutants of Neurospora crassa. Genetics. 75(4):605-13.

Gardner, G.F., Feldman, J.F., 1980. The frq locus in Neurospora crassa: a key element in circadian clock organization. Genetics. 96, 877-886.

Gin, E., Diernfellner, A.C., Brunner, M., Höfer, T., 2013. The Neurospora photoreceptor VIVID exerts negative and positive control on light sensing to achieve adaptation. Mol. Syst. Biol. 9, 667.

Glossop, N.R., Lyons, L.C., Hardin, P.E., 1999. Interlocked feedback loops within the Drosophila circadian oscillator. Science 286, 766-768.

Goldbeter, A., 1995. A model for circadian oscillations in the Drosophila period protein (PER). Proc. Biol. Sci. 261, 319-324.

Gonze, D., Goldbeter, F.A., 2000. Entrainment versus chaos in a model for a circadian oscillator driven by light-dark cycles. J. Statist. Phys. 101, 649-663.

Gonze, D., Leloup, J.-C., Goldbeter, A., 2000 Theoretical models for circadian rhythms in Neurospora and Drosophila. C R Acad. Sci. III 323, 57-67.

He, Q., Cha, J., He, Q., Lee, H.-C., Yang, Y., Liu, Y., 2006. CKI and CKII mediate the FREQUENCY-dependent phosphorylation of the WHITE COLLAR complex to close the Neurospora circadian negative feedback loop. Genes Dev. 20, 2552-2565.

Hunt, S.M., Thompson, S., Elvin, M., Heintzen, C., 2010. VIVID interacts with the WHITE COLLAR complex and FREQUENCY-interacting RNA helicase to alter light and clock responses in Neurospora. Proc. Natl. Acad. Sci. U S A 107, 16709-16714.

Malzahn, E., Ciprianidis, S., Káldi, K., Schafmeier, T., Brunner, M., 2010. Photoadaptation in Neurospora by competitive interaction of activating and inhibitory LOV domains. Cell 142, 762-772.

Nsa, I.Y., Karunarathna, N., Liu, X., Huang, H., Boetteger, B., Bell-Pedersen, D., 2015. A novel cryptochrome-dependent oscillator in Neurospora crassa. Genetics 199, 233-245.

Schafmeier, T., Haase, A., Káldi, K., Scholz, J., Fuchs, M., Brunner, M., 2005. Transcriptional feedback of Neurospora circadian clock gene by phosphorylation-dependent inactivation of its transcription factor. Cell 122, 235-246.

Trapnell, C., Williams, B.A., Pertea, G., Mortazavi, A., Kwan, G., van Baren, M. J., Salzberg, S. L., Wold, B. J., Pachter, L., 2010. Transcript assembly and quantification by RNA-Seq reveals unannotated transcripts and isoform switching during cell differentiation. Nat. Biotechnol. 28, 511–515.
